# Patent Medicines

**Published:** 1857-12

**Authors:** 


### PATENT MEDICINES.

"It is estimated that * * * pills have done more to promote the public health than any other cause. There can be no doubt that these medicines have very much reduced the proportion of deaths from consumptive diseases in this country."

The above we find in a religious newspaper, among the items of "News of the Week," and placed, not among the advertisements of the paper, but immediately over the notices of marriages and deaths, consequently in the most noticeable part of the paper.

Here are religious men, who for pay lend their paper, week after week, to the statement of a demonstrable falsehood. They know that these medicines have *not* "done more to promote the public health than any other cause." The second deception practiced upon its readers is, the placing the statement among the news items of the week, and receiving pay for the same.

Is the statement either new or true? And so far from these medicines "having very much reduced the proportion of deaths from consumptive diseases in this country," the evidence of published statements is, that a greater number of deaths from consumption are occurring, according to the population, than heretofore. Thus it is that religious men, in their public and official capacities, are obliterating the lines of distinction which should separate them from men of the world. They thus sell their principles and lend themselves to the dissemination of falsehoods, for a price.

